# Receiving support to quit smoking and quit attempts among smokers with and without smoking related diseases: Findings from the EUREST-PLUS ITC Europe Surveys

**DOI:** 10.18332/tid/102787

**Published:** 2019-03-20

**Authors:** Linnea Hedman, Paraskevi A. Katsaounou, Filippos T. Filippidis, Sofia B. Ravara, Anne Lindberg, Christer Janson, Christina Gratziou, Gernot Rohde, Christina N. Kyriakos, Ute Mons, Esteve Fernández, Antigona C. Trofor, Tibor Demjén, Krzysztof Przewoźniak, Yannis Tountas, Geoffrey T. Fong, Constantine I. Vardavas, on behalf of the EUREST-PLUS consortium

**Affiliations:** 1 The Tobacco Control Committee of the European Respiratory Society, Lausanne, Switzerland; 2 Division of Occupational and Environmental Medicine, Department of Public Health and Clinical Medicine, The OLIN Unit, Umeå University, Umeå, Sweden; 3 National and Kapodistrian University of Athens (UoA), Athens, Greece; 4 Department of Primary Care and Public Health, Imperial College, London, United Kingdom; 5 Health Sciences Research Centre (CICS-UBI), Faculty of Health Sciences, University of Beira Interior, Covilha, Portugal; 6 Public Health Research Centre, National School of Public Health, NOVA University, Lisbon, Portugal; 7 Department of Medical Sciences, Uppsala University, Uppsala, Sweden; 8 Medical Clinic I, Department of Respiratory Medicine, Goethe University Hospital, Frankfurt, Germany; 9 European Network for Smoking and Tobacco Prevention (ENSP), Brussels, Belgium; 10 University of Crete (UoC), Heraklion, Greece; 11 Cancer Prevention Unit and WHO Collaborating Centre for Tobacco Control, German Cancer Research Center (DKFZ), Heidelberg, Germany; 12 Institut Català d’Oncologia and Bellvitge Biomedical Research Institute (IDIBELL), Catalonia, Spain; 13 School of Medicine and Health Sciences, Universitat de Barcelona, Catalonia, Spain; 14 University of Medicine and Pharmacy ‘Grigore T. Popa’ Iasi, Iasi, Romania; 15 Aer Pur Romania, Bucharest, Romania; 16 Smoking or Health Hungarian Foundation (SHHF), Budapest, Hungary; 17 Health Promotion Foundation (HPF), Warsaw, Poland; 18 Maria Skłodowska-Curie Institute-Oncology Center (MSCI), Warsaw, Poland; 19 Department of Psychology and School of Public Health and Health Systems, University of Waterloo (UW), Waterloo, Canada; 20 Ontario Institute for Cancer Research, Toronto, Canada; * Contributed equally

**Keywords:** smoking cessation, chronic disease, Chronic Obstructive Pulmonary Disease (COPD), quitting smoking, tobacco smoking

## Abstract

**INTRODUCTION:**

Having a chronic disease either caused or worsened by tobacco smoking does not always translate into quitting smoking. Although smoking cessation is one of the most cost-effective medical interventions, it remains poorly implemented in healthcare settings. The aim was to examine whether smokers with chronic and respiratory diseases were more likely to receive support to quit smoking by a healthcare provider or make a quit attempt than smokers without these diseases.

**METHODS:**

This population-based study included a sample of 6011 adult smokers in six European countries. The participants were interviewed face-to-face and asked questions on sociodemographic characteristics, current diagnoses for chronic diseases, healthcare visits in the last 12 months and, if so, whether they had received any support to quit smoking. Questions on smoking behavior included nicotine dependence, motivation to quit smoking and quit attempts in the last 12 months. The results are presented as weighted percentages with 95% confidence intervals (CI) and as adjusted odds ratios with 95% CI based on logistic regression analyses.

**RESULTS:**

Smokers with chronic respiratory disease, those aged 55 years and older, as well as those with one or more chronic diseases were more likely to receive smoking cessation advice from a healthcare professional. Making a quit attempt in the last year was related to younger age, high educational level, higher motivation to quit, lower nicotine dependence and having received advice to quit from a healthcare professional but not with having chronic diseases. There were significant differences between countries with smokers in Romania consistently reporting more support to quit as well as quit attempts.

**CONCLUSIONS:**

Although smokers with respiratory disease did indeed receive smoking cessation support more often than smokers without disease, many smokers did not receive any advice or support to quit during a healthcare visit.

## INTRODUCTION

Five respiratory conditions, coined the ‘big five’ — Chronic Obstructive Pulmonary Disease (COPD), asthma, acute respiratory infections, tuberculosis and lung cancer — are a great burden on society and rank among the top eight causes of mortality^1,2^. All of these conditions as well as several other chronic diseases such as cardiovascular disease, diabetes, rheumatoid arthritis and cancer of most organs, are either caused or worsened by tobacco smoking^3,4^. For smokers who have these diseases, quitting is paramount to improving their health^3,5-7^.

However, having chronic diseases or multiple comorbidities caused or worsened by smoking does not always translate into quitting smoking. On the contrary, many patients with chronic diseases are more reluctant to quit, regardless of their disease^6,8,9^. The lack of motivation could be attributed to the intensity of nicotine addiction, but also to the lack of personalized advice and motivational interview skills from healthcare providers.

Smoking behaviour variables, such as motivation to quit and nicotine dependence, are crucial determinants for both quit attempts and success rates^10^. While motivation to quit is central to fostering quit attempts, the success of the attempt is strongly related to the level of nicotine dependence^8^. It has been shown that smokers with chronic diseases, such as COPD or depression, have higher nicotine dependence than smokers without these diseases^11,12^. Thus, smokers with chronic diseases may experience more difficulties quitting compared to the general population of smokers and therefore need tailored assistance^6,13^. Again, even if smokers are making quit attempts they may fail without adequate professional assistance.

Although evidence-based smoking cessation is one of the most cost-effective medical interventions, it remains poorly implemented in healthcare settings and is not yet integrated in standard medical care^6,7,14^. Physicians often do not routinely provide evidence-based smoking cessation treatment to their patients mainly due to barriers such as frustration, negative attitudes towards patients who continue to smoke, and lack of experience with smoking cessation techniques^15^.

Current research suggests that physician assistance in smoking cessation (i.e. brief counseling and pharmacotherapy) may be increased among smokers with chronic diseases than smokers without diseases^16-18^. Amongst sociodemographic factors that have been found to influence smoking behavior and cessation assistance are age, income, and educational level^19-21^. However, few studies have explored the presence of diseases and sociodemographic factors on both provider-level and patient-level outcomes^22,23^, and even fewer among European population cohorts. The existence of cross-country differences in the implementation of smoking cessation policies across Europe further highlights the need for examining how physician assistance in smoking cessation differs across countries, which has significant implications for context-specific interventions.

The aim of the current study was to examine whether smokers with chronic and respiratory diseases were more likely to receive support to quit smoking by a healthcare provider or make a quit attempt than smokers without these diseases. Furthermore, we investigate cross-country differences and other sociodemographic factors associated with receiving support to quit and quit attempts among smokers in six European Union (EU) Member States. Findings from this study could have significant implications for tailoring interventions to more effectively address smoking cessation in some of the most high-risk groups of smokers.

## METHODS

### Design

The current study is part of a European Commission Horizon-2020 funded study entitled *European Regulatory Science on Tobacco: Policy implementation to reduce lung diseases* (EUREST-PLUS-HCO-06-2015). The aim of the EUREST-PLUS H2020 is to monitor and evaluate the impact of tobacco control policies at a European level within the context of the newly implemented Tobacco Products Directive (TPD) (2014/40/EU) and the World Health Organization (WHO) Framework Convention on Tobacco Control (FCTC)^24,25^.

Using the methodology of the International Tobacco Control Evaluation (ITC) Project, the EUREST-PLUS ITC Wave 1 Survey was conducted between 18 June and 12 September 2016 among 6011 nationally representative adult cigarette smokers aged 18 years and older (about 1000 in each of Germany, Greece, Hungary, Poland, Romania and Spain). The geographic strata were Nomenclature of Territorial Units for Statistics (NUTS) regions crossed with degree of urbanization (urban, intermediate, rural). Approximately 100 area clusters were sampled in each country. The aim was to obtain 10 adult smokers per cluster. Clusters were allocated to strata proportionally to population size (aged ≥18 years). Within each cluster, household addresses were sampled using a random walk design. One randomly selected male smoker and one randomly selected female smoker were chosen for interview from a sampled household, where possible. Screening of households continued until the required number of smokers from the cluster had been interviewed. All interviews were conducted face-to-face by interviewers using tablets (Computer Assisted Personal Interviewing, CAPI). Additional details on the methods of EUREST-PLUS ITC 6E are described elsewhere^25^.

### Measures

All participants were asked if they had visited a healthcare professional in the past 12 months. If they had, they were further asked if they received: a) advice to quit smoking; b) additional help or a referral to another service to help you quit; c) a prescription for stop-smoking medication (medication was defined as pills, nicotine patches and nicotine gum; or d) self-help cessation aids such as pamphlets or brochures during any visit to a doctor or health professional in the past 12 months. By responding ‘yes’ to any of these questions, they were considered as having received support to quit smoking, and by responding ‘yes’ to b) or c), they were considered as having received referral or medications to quit by a healthcare professional in the past 12 months. We have separately studied the outcome ‘having received either referral or Rx to quit’, that involves methods that have been shown to be more efficient than all others^26^.

Participants were asked: ‘are you currently being treated for, or do you have a current diagnosis for, any of the following? — severe obesity; depression; anxiety; alcohol problems; chronic pain; diabetes; cancer, excluding non-melanoma skin cancer; heart disease; chronic lung disease such as emphysema, chronic bronchitis etc.’. Based on their responses, participants were classified either by number or type of disease:

- Number of chronic diseases
  1. No disease
  2. Any disease
  3. Two or more diseases
- Disease type
  1. Any chronic respiratory disease
  2. Any chronic non-respiratory disease

Among the included mental illnesses, depression was the most common. Because it is strongly correlated with increasing smoking rates, we also performed the analyses separately for depression.

Past quit attempts were assessed with the question: ‘have you made an attempt to quit smoking in the last 12 months?’ with potential responses including ‘yes’, ‘no’ and ‘don’t know’.

Sociodemographic characteristics assessed included age (18–24; 25–39; 40–54; and ≥55 years), sex (male, female); education (low: primary school; moderate: upper secondary school; high: higher education); income (low, moderate, high, not reported); marital status (married/cohabiting, single, widowed/ divorced/separated); and country of residence. The threshold for income level varied between the countries due to differences in, for instance, levels of salary and general cost for living (Supplementary Table 1).

Nicotine dependence was assessed by the measure of the time to first cigarette, with the question: ‘On days that you smoke, how soon after waking do you usually have your first smoke?’ (>30 minutes; 6–30 minutes; ≤5 minutes).

Motivation to quit was measured with two questions. First, participants were asked: ‘Are you planning to quit smoking…?’ (within the next month; within the next 6 months; sometime in the future; beyond 6 months; not planning to quit). Those who responded not planning to quit were classified as having no motivation to quit. Those who said they plan to quit were asked the question: ‘How much do you want to quit…?’ (a little; somewhat; a lot). The motivation to quit variable is divided into four categories: no motivation; a little; somewhat; a lot.

### Analysis

We present percentages and 95% confidence intervals (CI) of participants having visited a physician or healthcare professional during the past 12 months and separately having attempted to quit during the same period. We also present weighted percentages of having received support to quit among those who have visited a healthcare professional in the past 12 months. Statistical significance of differences between categories was based on non-overlapping confidence intervals. We ran a logistic regression model to assess the association of having attempted to quit with age, sex, income, education, marital status, number of diseases and country of residence, as well as nicotine dependence and motivation to quit smoking. A similar model was fitted among those who had visited a healthcare professional in the past 12 months to assess the association of having received support to quit by a healthcare professional with the same sociodemographic factors. Model specification was determined by comparing Bayesian Information Criterion (BIC) and Akaike Information Criterion (AIC). Logistic regression results are presented as adjusted odds ratios (aOR) with 95% CI. In all analyses we used weights to account for the complex sampling of the surveys and the procedure has previously been described in detail^27^. Briefly, we used bootstrap weights that correct and adjust for sample misrepresentation due to factors such as unequal sampling probabilities, frame error, and non-responses, which resulted in samples representative at the country level in terms of age, sex and region of residence. All analyses were conducted with Stata 14.0.

## RESULTS

Sociodemographic characteristics of the study sample are presented in Supplementary Table 1. In short, the age and sex distribution as well as marital status were similar in the six countries. Having low educational level was most common in Hungary (65%) and least common in Poland (12%), whereas high educational level was most common in Greece (21%) and least common in Hungary (6%).

### Smoking behavior

As presented in Table 1, participants were predominantly daily smokers, ranging from 88.3% in Germany to 98.9% in Hungary. The lowest nicotine dependence was found in Germany where 47.5% smoked their first cigarette more than half an hour after waking up. The highest dependence was found in Romania where 34.3% smoked their first cigarette less than 5 minutes after waking up. At the same time, Romania had the highest proportion of smokers with ‘a lot’ of motivation to quit (28.7%) (Table 2). Overall, 57% had no motivation to quit and the country with the highest prevalence of participants with no motivation to quit was Hungary with 68%.

**Table 1:** The prevalence of daily smoking and nicotine dependence among smokers in six European countries

*Country*	*Sample size*	*Daily smokers % (95% CI)*	*Nicotine dependence (minutes to first cigarette after waking up)*		
			*≥31 % (95% CI)*	*6–30 % (95% CI)*	*≤5 % (95% CI)*
Germany	1003	88.3 (85.3–91.4)	47.5 (42.3–52.7)	38.6 (34.2–43.0)	14.0 (10.8–17.1)
Greece	1000	96.9 (95.2–98.6)	25.3 (21.2–29.4)	49.6 (44.2–55.1)	25.0 (21.2–28.9)
Hungary	1000	98.9 (98.2–99.7)	21.2 (17.2–25.1)	49.2 (44.9–53.4)	29.7 (24.9–34.4)
Poland	1006	96.4 (95.3–97.4)	27.9 (24.0–31.8)	47.0 (42.5–51.6)	25.1 (20.9–29.2)
Romania	1001	94.8 (93.0–96.6)	21.5 (18.1–24.9)	44.2 (40.5–47.8)	34.3 (30.0–38.7)
Spain	1001	97.2 (96.0–98.3)	42.0 (37.8–46.3)	37.5 (33.7–41.3)	20.5 (16.8–24.2)
Overall	6011		30.7 (29.0–32.4)	44.4 (42.7–46.2)	24.9 (23.2–26.6)

**Table 2:** Motivation to quit smoking among smokers in six European countries

*Country*	*No motivation to quit % (95% CI)*	*A little % (95% CI)*	*Somewhat % (95% CI)*	*A lot % (95% CI)*
Germany	42.6 (37.4–47.8)	18.5 (15.8–21.2)	24.0 (21.0–26.9)	14.9 (11.5–18.3)
Greece	59.5 (55.4–63.7)	8.2 (5.7–10.6)	19.8 (16.9–22.6)	12.6 (9.7–15.4)
Hungary	68.1 (64.1–72.0)	6.1 (3.3–8.8)	17.6 (14.4–20.8)	8.3 (6.0–10.6)
Poland	58.9 (54.4–63.5)	5.1 (3.3–6.9)	23.4 (19.8–26.9)	12.6 (9.6–15.6)
Romania	46.4 (40.8–52.1)	5.4 (3.7–7.1)	19.5 (15.6–23.3)	28.7 (24.3–33.1)
Spain	63.5 (58.9–68.0)	10.1 (7.5–12.7)	14.1 (10.7–17.4)	12.4 (9.8–15.0)
Overall	56.5 (54.5–58.4)	9.0 (8.0–10.0)	19.6 (18.3–21.0)	15.0 (13.7–16.3)

### The prevalence of chronic diseases

The prevalence of having any disease was highest in Germany (26.2%) and lowest in Romania (13.3%) (Table 3). Germany also reported the highest prevalence of having two or more diseases (10.3%) and Greece having the lowest (4.4%). Having any chronic respiratory disease did not vary by country. Similarly, smokers in Germany reported the highest prevalence of depression (5.2%) and Greece the lowest (1.9%).

**Table 3:** Prevalence of chronic respiratory disease, any chronic disease, number of diseases and depression among smokers in six European countries

*Country*	*Any disease % (95% CI)*	*Two or more diseases % (95% CI)*	*Any chronic respiratory disease % (95% CI)*	*Depression % (95% CI)*
Germany	26.2 (23.3–29.1)	10.3 (8.1–12.4)	3.7 (2.5–4.8)	5.2 (3.4–6.9)
Greece	15.8 (13.0–18.6)	4.4 (2.9–6.0)	3.2 (2.1–4.3)	1.9 (0.8–3.0)
Hungary	17.7 (14.1–21.4)	8.8 (6.0–11.6)	3.4 (1.9–4.8)	4.4 (2.1–6.7)
Poland	15.1 (12.2–18.0)	6.0 (3.9–8.2)	4.6 (2.7–6.4)	3.3 (1.7–4.9)
Romania	13.3 (10.7–16.0)	5.5 (3.7–7.2)	2.3 (1.4–3.1)	2.6 (1.6–3.7)
Spain	18.8 (15.2–22.4)	6.4 (4.2–8.6)	3.9 (2.8–4.9)	4.3 (2.2–6.5)
Overall	17.8 (16.5–19.2)	6.9 (6.0–7.8)	3.5 (3.0–4.0)	3.6 (2.9–4.3)

### Support to quit smoking by a health professional and quit attempts

The prevalence of visiting a doctor or health professional over the past 12 months was lowest in Greece (30.6%) and highest in Spain (56.1%) (Table 4). Among those who had visited a doctor, 60.8% of participants from Romania and 53.3% in Greece had received any support or advice to quit smoking over the past 12 months, with the lowest percentages reported in Poland (23.0%) and Hungary (24.5%). The most commonly reported type of support to quit smoking was receiving advice, with similar cross-country differences observed as receiving any advice or support. Receiving pamphlets was the next most common type of assistance reported, ranging from 16.5% (Romania) to 5.1% (Greece). The prevalence of those who received a referral was also highest in Romania (8.6%). Receiving a prescription was the least commonly reported type of support, with no observations in Germany and the highest percentage reported in Poland (3.0%), with little variation between countries. The percentage of participants reporting having made a quit attempt in the past 12 months was highest in Romania (27.1%) and lowest in Hungary (10.4%).

**Table 4:** Advice and other support to quit smoking during any visit to the doctor or health professional and quit attempts over the past 12 months

*Country*	*Visited doctor over past 12 months % (95% CI)*	Received any advice/ support* % (95% CI)	Received advice* % (95% CI)	Received referral* % (95% CI)	Received quitting prescription* % (95% CI)	Received pamphlets* % (95% CI)	*Quit attempt in past 12 months % (95% CI)*
Germany	47.6 (42.4–52.7)	41.2 (35.4–46.9)	39.3 (33.5–45.2)	2.3 (0.7–3.8)	No observations	10.7 (7.3–14.1)	17.1 (14.2–19.9)
Greece	30.6 (25.4–35.7)	53.3 (42.8–63.8)	53.0 (42.6–63.5)	2.8 (1.0–4.6)	2.0 (0.6–3.5)	5.1 (2.8–7.5)	15.1 (12.3–17.9)
Hungary	41.3 (35.9–46.8)	24.5 (18.9–30.2)	21.7 (16.3–27.1)	3.0 (1.2–4.7)	2.0 (0.7–3.3)	10.5 (6.9–14.1)	10.4 (8.2–12.6)
Poland	40.2 (35.0–45.3)	23.0 (18.2–27.9)	20.8 (16.4–25.1)	4.3 (2.0–6.6)	3.0 (0.5–5.4)	7.2 (4.3–10.0)	16.2 (13.2–19.3)
Romania	39.2 (35.5–42.8)	60.8 (56.1–65.6)	56.5 (51.4–61.5)	8.6 (4.6–12.5)	**	16.5 (10.8–22.1)	27.1 (23.9–30.2)
Spain	56.1 (50.4–61.7)	47.2 (41.5–52.8)	45.7 (39.8–51.6)	4.6 (2.5–6.7)	2.9 (1.4–4.3)	7.3 (4.6–10.0)	17.7 (14.2–21.1)
Overall	42.5 (40.4–44.6)	41.4 (38.7–44.2)	39.2 (36.5–42.0)	4.2 (3.3–5.2)	2.0 (1.3–2.6)	9.6 (8.0–11.1)	17.2 (16.1–18.4)

* Among those who visited a doctor (Germany: n=491; Greece: n=301; Hungary: n=430; Poland: n=420; Romania: n=407; Spain: n=551)

** Prescription medication for smoking cessation was not available in Romania and were therefore excluded.

### Receiving support to quit and quit attempts by disease type

Table 5 presents support to quit and quit attempts in the last 12 months by number and type of disease. Receiving support to quit smoking was statistically significantly more common among smokers with any chronic respiratory disease compared to no disease in all countries except Greece. Except for depression, the proportion of smokers with diseases receiving support to quit was highest in Romania. For instance, 95.0% of those with any chronic respiratory disease in Romania reported receiving support to quit compared to 57.2% in Poland and 64.7% in Hungary. Although the pattern was similar in all countries except Greece, Romania was the only country where the prevalence of quit attempts was statistically significantly higher in those with any chronic respiratory disease (49.6%) than those with no disease (25.7%). Smokers with depression received more support to quit in Greece and Spain but this did not result in more quit attempts.

**Table 5:** Support to quit smoking during any visit to the doctor or health professional, and quit attempts over the past 12 months by number and type of disease

*Country*	Received support to quit smoking during visit to doctor/health professionals*					*Depression % (95% CI)*
	*No disease % (95% CI)*	*Any disease % (95% CI)*	*Two or more diseases % (95% CI)*	*Any chronic respiratory disease % (95% CI)*	*Any non– respiratory disease % (95% CI)*	
Germany	32.3 (25.4–39.2)	55.7 (48.1–63.3)	54.7 (42.8–66.5)	77.9 (64.0–91.7)	54.7 (47.0–62.4)	45.3 (28.8–61.9)
Greece	48.9 (36.8–61.1)	65.4 (53.9–76.8)	76.9 (57.9–95.9)	76.8 (52.0–100.0)	65.5 (53.0–77.9)	78.7 (48.4–100.0)
Hungary	20.0 (14.1–25.9)	36.4 (27.3–45.4)	44.4 (30.3–58.4)	64.7 (47.8–81.7)	34.7 (25.3–44.1)	37.1 (14.0–60.2)
Poland	16.9 (11.5–22.4)	39.3 (28.2–50.4)	61.6 (45.8–77.3)	57.2 (30.8–83.5)	39.9 (28.4–51.4)	44.5 (21.0–68.0)
Romania	56.0 (50.7–61.3)	76.7 (67.7–85.8)	80.9 (61.2–100.0)	95.0 (87.9–100.0)	74.9 (65.0–84.7)	48.7 (17.7–79.6)
Spain	41.0 (35.4–46.5)	62.3 (50.7–73.9)	76.0 (62.8–89.3)	72.0 (58.1–85.8)	63.5 (50.4–76.5)	61.1 (39.8–82.3)
Overall	35.5 (32.5–38.5)	56.4 (51.9–60.8)	64.0 (57.5–70.5)	73.4 (65.9–80.9)	55.7 (51.0–60.4)	51.9 (41.5–62.3)
Germany	16.3 (13.0–19.6)	19.1 (14.6–23.6)	14.8 (8.4–1.2)	28.7 (18.1–39.4)	18.7 (14.1–23.2)	18.2 (9.5–26.8)	
Greece	15.4 (12.7–18.2)	13.4 (6.9–19.9)	22.2 (9.5–34.8)	13.3 (4.6–21.9)	14.4 (7.1–21.7)	19.2 (2.0–36.4)	
Hungary	9.7 (7.3–12.2)	14.4 (9.4–19.4)	16.9 (8.1–25.7)	11.1 (2.3–20.0)	13.9 (8.7–19.0)	20.1 (7.5–32.7)	
Poland	15.4 (12.4–18.4)	20.3 (14.4–26.2)	26.4 (17.7–35.0)	29.0 (18.1–40.0)	19.7 (14.1–25.3)	23.4 (14.5–32.4)	
Romania	25.7 (22.3–29.0)	36.3 (26.2–46.4)	30.4 (18.6–42.1)	49.6 (32.4–66.8)	34.6 (23.6–45.5)	25.2 (8.4–42.0)	
Spain	17.5 (13.3–21.7)	18.2 (12.7–23.7)	20.2 (11.3–29.1)	23.2 (11.5–35.0)	18.5 (12.6–24.3)	14.9 (3.9–25.8)	
Overall	16.8 (15.5–18.1)	19.6 (17.2–22.0)	20.6 (17.0–24.2)	24.9 (20.1–29.7)	19.4 (16.8–21.9)	19.6 (14.9–24.3)	

* Among respondents who have visited a healthcare professional (n=2300). ** Among all respondents (n=6011).

### Factors associated with making a quit attempt in the past 12 months

Among all smokers, compared to those aged 18–24 years, smokers aged 40–54 and ≥55 years were less likely to have made a quit attempt (Table 6). Smokers with a little, somewhat, and a lot of motivation to quit smoking, were all significantly more likely to make a quit attempt compared with smokers with no motivation to quit. Smokers reporting ≤5 minutes until smoking the first cigarette were significantly less likely to make a quit attempt. Respondents from Romania were 1.7 times more likely to report a quit attempt compared to those from Germany. Sex, income level, educational level, marital status, and number of diseases, were not associated with having made a quit attempt.

**Table 6:** Factors associated with receiving support to quit from a doctor/health professional and with having attempted to quit in the past 12 months, analyses were adjusted for sex, income, and marital status

*Factors*	Having made a quit attempt* aOR (95% CI)	Having made a quit attempt** aOR (95% CI)	Having received any support to quit** aOR (95% CI)	Having received referral or Rx to quit** aOR (95% CI)
**Age group (years)**				
18–24 (ref)				
25–39	0.75 (0.55–1.02)	0.84 (0.55–1.27)	1.00 (0.68–1.47)	1.35 (0.46–3.98)
40–54	0.49 (0.34–0.68)	0.49 (0.31–0.79)	1.38 (0.95–1.99)	2.03 (0.70–5.92)
≥55	0.53 (0.37–0.75)	0.59 (0.38–0.91)	1.75 (1.18–2.59)	1.56 (0.48–5.09)
**Education**				
Low (ref)				
Moderate	0.95 (0.77–1.18)	1.14 (0.88–1.48)	0.80 (0.63–1.02)	0.87 (0.55–1.38)
High	1.24 (0.92–1.68)	1.52 (1.05–2.21)	0.87 (0.62–1.23)	0.97 (0.45–2.08)
**Number of diseases**				
None (ref)				
One	1.10 (0.82–1.48)	0.77 (0.54–1.09)	1.69 (1.35–2.13)	0.87 (0.53–1.44)
Two or more	1.22 (0.91–1.64)	1.04 (0.71–1.50)	3.09 (2.25–4.24)	2.90 (1.76–4.79)
**Motivation to quit**				
No motivation to quit (ref)				
A little	1.91 (1.35–2.69)	1.31 (0.79–2.18)		
Somewhat	3.81 (3.00–4.82)	3.96 (2.98–5.26)		
A lot	10.92 (8.67–13.75)	8.07 (5.88–11.10)		
**Nicotine dependence**				
(minutes to first cigarette)				
>30 (ref)				
6–30	0.87 (0.72–1.05)	0.94 (0.74–1.20)		
≤5	0.70 (0.56–0.88)	0.66 (0.48–0.91)		
**Having received advice to quit**				
No (ref)				
Yes		1.43 (1.12–1.82)		
**Country**				
Germany (ref)				
Greece	0.79 (0.58–1.08)	1.37 (0.82–2.28)	1.98 (1.19–3.28)	1.59 (0.60–4.23)
Hungary	0.58 (0.42–0.81)	1.09 (0.67–1.77)	0.47 (0.31–0.71)	1.63 (0.69–3.82)
Poland	0.90 (0.68–1.21)	1.37 (0.86–2.19)	0.50 (0.34–0.75)	2.63 (1.10–6.28)
Romania	1.70 (1.31–2.22)	1.72 (1.10–2.69)	3.08 (2.18–4.34)	4.63 (1.91–11.26)
Spain	1.02 (0.74–1.39)	1.69 (1.09–2.62)	1.53 (1.07–2.19)	2.77 (1.28–6.02)

* Amongst all respondents (n=6011).

** Amongst respondents who have visited a healthcare professional (n=2300).

The corresponding adjusted analysis was performed among the smokers that had made a healthcare visit in the last 12 months (n=2300) and the results are presented in Table 6. Those who had received advice to quit were significantly more likely to have made a quit attempt. Having made a quit attempt was also significantly associated with high educational level, compared to a low educational level.

### Factors associated with receiving quit support from a doctor or health professional in the past 12 months

Among the 2300 smokers who had made a healthcare visit in the last 12 months, those aged ≥55 years were significantly more likely to have received support to quit compared to those of age 18–24 years (Table 6). Women were significantly less likely to have received support to quit than men. Compared to those with no disease, those with one disease were 1.69 times as likely (aOR=1.69; 95% CI: 1.35–2.13) to have received support to quit, and those with two or more diseases were 3.09 times as likely (aOR=3.09; 95% CI: 2.25–4.24) to have received support. The probability of having received support to quit was significantly higher in Romania, Greece and Spain, compared to Germany. Having received referral or medication to quit was significantly related to having two or more diseases, and living in Poland, Romania or Spain. Income, educational level and marital status were not associated with having received support.

## DISCUSSION

In this population-based sample of smokers in six European countries, we found that smokers with chronic respiratory disease, those aged ≥55 years, as well as those with one or more chronic diseases were more likely to receive smoking cessation advice from their physician, compared to smokers with no disease. Making a quit attempt in the last year was related to younger age, high educational level, higher motivation to quit, lower nicotine dependence and having received advice to quit from a healthcare professional but not with having chronic diseases. There were significant differences between countries with smokers in Romania consistently reporting more support to quit as well as quit attempts.

Smokers with chronic respiratory diseases did indeed receive smoking cessation support more often than smokers without diseases. It seems that the existence of chronic respiratory disease may increase physician engagement in cessation as smokers with respiratory disease received support more often than smokers with other chronic non-respiratory diseases, in accordance with other studies^16-18^. Smokers with respiratory disease may contemplate quitting smoking and therefore request advice and support from healthcare professionals more often than smokers without any diseases. However, respiratory patients may be more socially disadvantaged and struggle more to quit smoking^6,7^. Moreover, smoking is common among people diagnosed with psychiatric disorders and have smoking rates substantially higher than the general population^28^. Although a high proportion of smokers with depression in our study had received advice for smoking cessation in most of the countries (37% to 79%) it seems that this was not sufficient to prompt a quit attempt. Smokers with psychiatric disorders find it most difficult to quit and hence they are a population group that needs intense smoking cessation support by trained staff.

Nevertheless, smoking cessation should be a primary objective of medical treatment of patients who smoke, regardless of existing or developing health problems. Screening smokers in routine clinical practice in combination with more comprehensive support such as referral to a follow-up or treatment combining behavioral counseling and pharmacotherapy may increase the quit success rates^14^. In our study, the proportion of smokers who visited a doctor and received advice to quit smoking varied from 21% to 57% across the different countries. Unfortunately, most physicians and other healthcare providers have limited training in smoking cessation skills, both at undergraduate and graduate level^29,30^. Although we did not assess reasons for not receiving support to quit, in a study where physicians in seven different countries were interviewed on their engagement in smoking cessation treatment, some of the barriers described were lack of time, limited knowledge and experience or skills^15^. Still, even brief advice may increase the motivation to quit^10^ and this simple method should be included in medical education and more consistently implemented in healthcare. The inadequacy of health professionals’ training in tobacco cessation is a critical health system weakness resulting in inconsistent delivery of tobacco cessation brief advice by healthcare providers as well as low availability of tobacco cessation services in many countries. The FCTC Article 14 emphasizes supporting current tobacco users to quit as a key component of a comprehensive tobacco control strategy to avert premature deaths in noncommunicable diseases. Although smoking cessation already has been incorporated in the training of respiratory medicine in the last eight years in most European Countries and in the HERMES syllabus, wider approaches incorporating the training of all healthcare providers in brief advice should be implemented^31^.

We found a dose-response association where the odds of both receiving smoking cessation support and having made a quit attempt increased with increasing number of chronic diseases, in accordance with other studies^32^. However, despite receiving support to quit smoking more often, there was no statistically significant difference in the prevalence of quit attempts between smokers with respiratory disease and smokers without diseases, in contrast to other studies^32^. The only exception was in Romania where, for instance, 50% of the smokers with a respiratory disease had made a quit attempt, compared to 26% among those without disease. Romania has recently implemented legislation banning smoking in public spaces, and since 2007 smoking cessation is available free-of-charge in a national governmentally funded program developed within lung disease clinics, which may have contributed to our findings^33^.

Continued smoking may exacerbate symptoms and accelerate the progression of many chronic diseases. Worries about future health problems may motivate smokers to quit smoking^34^ but other studies have shown that having COPD or respiratory symptoms was not enough to motivate a person to quit^35^. Moreover, it has also been demonstrated that smokers with more advanced COPD are often excluded from cessation interventions^36^. It is alarming that many smokers did not receive any advice or support to quit smoking during a visit to their physician. Only in two countries (Romania and Greece) more than half received any advice or cessation support. Among those who received support, the most frequent assistance was self-help aid (pamphlets), which has been shown to be a less effective type of cessation support, especially if it is not individually tailored^14^. Most recent data show that the majority of smokers in the EU attempt to quit without assistance and that the proportion of those who receive assistance from health professionals is decreasing^37^. Moreover, there may be sociodemographic factors related to the patient that influence the physicians’ engagement. It has been shown that smokers with higher socioeconomic status were more likely to receive smoking cessation support^19-21^. In our study, we found that smokers with high educational level were more likely to make a quit attempt than those with lower educational level. Furthermore, having made a quit attempt was more common among smokers of younger ages, lower nicotine dependence and higher motivation, consistent with the literature^8,38^. Healthcare providers should motivate and engage younger smokers to quit, since they often are less dependent, have more chances of quitting, and obtain greater health gains.

Thus, there is a great challenge to motivate smokers to quit and support them in developing strong determination to succeed but there is no ‘one size fits all’ approach. Interventions need to be specifically tailored in order to more effectively address smoking cessation in the most high-risk groups of smokers, irrespective of their mental health or socioeconomic status. Providing smoking cessation support and treatment to patients with chronic diseases are not only important for reducing the risk for complications but also to reduce the risk for developing other smoking induced diseases.

### Limitations and strengths

Some of the strengths of our study are the use of a large population-based sample of smokers plus validated and reliable methods. There are also some limitations, for example, all four interventions (‘advice to quit smoking’; ‘additional help or a referral to another service to help you quit’; ‘a prescription for stop-smoking medication’ or ‘pamphlets or brochures on how to quit’) were considered as proxies for having received support, although they have very different efficacy rates^26^. Both information on diseases, advice to quit and quit attempts were self-reported and may be subject to reporting or recall bias. Also, there may be differences in the health systems between the six countries, as some of them may be more thorough in investigating and diagnosing potential diseases. Moreover, there may be differences in the training of healthcare professionals in smoking cessation and brief advice. Lastly, there is variation in the prevalence of smoking (Greece 37%, Romania 28%, Germany 25%, Hungary 27%, Poland 30%, Spain 28%) and in successful quit attempts among countries, which may introduce selection bias^39^. Another limitation may be that our study sample excluded smokers that quit during the last 12 months, which may have resulted in a sample representing smokers that failed to quit. Finally, in our definition of number of chronic diseases we chose to include all types of diseases and did not perform the analyses separately for disease types (e.g. mental illness, heart disease, cancer, diabetes).

## CONCLUSIONS

Smokers with chronic respiratory diseases did indeed receive smoking cessation support more often than smokers without diseases and there was a dose-response association between a higher number of chronic diseases and the likelihood of receiving smoking cessation support and having made a quit attempt. Alarmingly, many smokers did not receive any advice or support to quit smoking during a visit to their physician. Thus, the engagement in smoking cessation among healthcare providers still seems to be a highly neglected task. Providing smoking cessation support and treatment to patients with chronic diseases are not only important for reducing the risk for complications but also to reduce the risk for developing other smoking induced diseases.

## CONFLICTS OF INTEREST

The authors declare that they have no competing interests, financial or otherwise, related to the current work. A Lindberg reports personal fees from Boehringer-Ingelheim, from AstraZeneca, from Novartis, and from Active Care, outside the submitted work. G Rohde reports personal fees from Pfizer, Boehringer Ingelheim, Solvay, GSK, Essex Pharma, MSD, Grifols, Chiesi, Vertex, Berlin Chemie, Astra-Zeneca, Bayer, Roche and Novartis for lectures including service on speakers’ bureaus outside the submitted work and/or consultancy during advisory board meetings and personal fees from GSK for travel accommodation/meeting expenses, outside the submitted work. K Przewoźniak reports grants and personal fees from Polska Liga Walki Z Rakiem (Polish League Against Cancer) outside the submitted work. CI Vardavas reports that he is the Strategic Development Editor of TID and that there are no conflicts of interest with this current work. The rest of the authors have also completed and submitted an ICMJE form for disclosure of potential conflicts of interest.
