# Supplementary material for: Receiving support to quit smoking and quit attempts among smokers with and without smoking related diseases: Findings from the EUREST-PLUS ITC Europe Surveys
Source: Tob Induc Dis. 2019 Mar 20;16:A14. doi: 10.18332/tid/102787 (PMC6661851; doi:10.18332/tid/102787)
Supplement: Supplementary file 1 [file TID-16-A14-s1.pdf]

Supplementary Table 1. Sample characteristics

		Germany n (weighted %)	Greece n (weighted %)	Hungary n (weighted %)	Poland n (weighted %)	Romania n (weighted %)	Spain n (weighted %)	Total n (weighted %)
Sex								
	Male	507 (60.9)	544 (53.2)	521 (59.2)	477 (55.4)	581 (58.2)	545 (57.3)	3175 (57.4)
	Female	496 (39.1)	456 (46.8)	479 (40.8)	529 (44.6)	420 (41.8)	456 (42.7)	2836 (42.6)
Age (years)								
	18–24	88 (8.4)	61 (8.4)	59 (9.3)	72 (8.0)	110 (14.3)	117 (12.2)	507 (10.1)
	25–39	283 (25.6)	255 (28.9)	282 (34.0)	342 (33.5)	300 (38.0)	312 (29.0)	1774 (31.5)
	40–54	339 (36.4)	383 (35.6)	357 (33.5)	281 (29.1)	321 (30.9)	323 (38.5)	2004 (34.0)
	≥55	293 (29.6)	301 (27.1)	302 (23.3)	311 (29.4)	270 (16.8)	249 (20.4)	1726 (24.4)
Income								
	Low	306 (29.8)	180 (18.3)	179 (16.1)	173 (15.5)	226 (19.5)	271 (26.9)	1335 (21.0)
	Moderate	347 (33.5)	525 (54.8)	290 (29.0)	353 (34.7)	466 (45.6)	268 (27.5)	2249 (37.5)
	High	257 (25.9)	98 (9.2)	220 (23.3)	154 (16.7)	250 (29.5)	68 (6.2)	1047 (18.4)
	Not reported	93 (10.8)	197 (17.7)	311 (31.6)	326 (33.1)	59 (5.5)	394 (39.4)	1380 (23.0)
Education								
	Low	509 (49.6)	306 (30.2)	617 (64.8)	123 (11.8)	246 (24.8)	410 (44.2)	2211 (37.7)
	Moderate	417 (42.4)	488 (48.9)	311 (29.1)	753 (77.4)	629 (62.9)	506 (47.9)	3104 (51.4)
	High	75 (7.9)	203 (20.8)	70 (6.1)	114 (10.8)	114 (12.3)	83 (7.9)	659 (11.0)
Marital status								
	Married/living with partner	601 (62.7)	695 (66.3)	664 (66.6)	670 (65.8)	704 (67.4)	592 (58.9)	3926 (64.6)
	Single	249 (24.1)	187 (23.1)	163 (19.8)	189 (22.2)	185 (22.9)	290 (29.0)	1263 (23.5)
	Divorced/widowed	153 (13.2)	116 (10.6)	170 (13.6)	135 (12.0)	111 (9.7)	119 (12.0)	804 (11.9)

© 2019 Hedman L.
